# Integrated Bioinformatics and Validation of lncRNA-Mediated ceRNA Network in Myocardial Ischemia/Reperfusion Injury

**DOI:** 10.1155/2022/7260801

**Published:** 2022-09-21

**Authors:** Ying Han, Gong Jin, Min Pan, Zhoufei Fang, Dan Lu, Wenqin Cai, Changsheng Xu

**Affiliations:** ^1^Department of Geriatrics, Hypertension Department, Fujian Hypertension Research Institute, Clinical Research Center for Geriatric Hypertension Disease of Fujian Province, Branch of National Clinical Research Center for Aging and Medicine, The First Affiliated Hospital of Fujian Medical University, Fuzhou, China; ^2^Department of General Practice, The First Affiliated Hospital of Fujian Medical University, Fuzhou, China; ^3^Fujian Hypertension Research Institute, The First Affiliated Hospital of Fujian Medical University, Fuzhou, China

## Abstract

**Background:**

Myocardial ischemia/reperfusion (MI/R) injury is a common pathology in ischemia heart disease. Long noncoding RNAs (lncRNAs) are significant regulators related to many ischemia/reperfusion conditions. This study is aimed at exploring the molecule mechanism of lncRNA-mediated competing endogenous RNA (ceRNA) network in MI/R.

**Methods:**

The dataset profiles of MI/R and normal tissues (GSE130217 and GSE124176) were obtained from the GEO database. Integrated bioinformatics were performed to screen out differentially expressed genes (DEGs). Thereafter, an lncRNA-mediated ceRNA network was constructed by the starBase database. The GO annotations and KEGG pathway analysis were conducted to study action mechanism and related pathways of DEGs in MI/R. A model of hypoxia/reoxygenation- (H/R-) treated HL-1 cell was performed to verify the expression of lncRNAs through qRT-PCR.

**Results:**

2406 differentially expressed- (DE-) mRNAs, 70 DE-lncRNAs, and 156 DE-miRNAs were acquired. These DEGs were conducted to construct an lncRNA-mediated ceRNA network, and a subnetwork including lncRNA Xist/miRNA-133c/mRNA (Slc30a9) was screen out. The functional enrichment analyses revealed that the lncRNAs involved in the ceRNA network might functions in oxidative stress and calcium signaling pathway. The lncRNA Xist expression is reduced under H/R conditions, followed by the increased level of miRNA-133c, thus downregulating the expression of Slc30a9.

**Conclusion:**

In sum, the identified ceRNA network which included the lncRNA Xist/miR-133c/Slc30a9 axis might contribute a better understanding to the pathogenesis and development of MI/R injury and offer a novel targeted therapy way.

## 1. Introduction

Ischemia-reperfusion injury (I/R) is a common pathology with high rates of death and hospitalization worldwide [1]. Notably, ischemia heart disease has increased prevalence and exacerbated myocardial infarction with aging [2]. Myocardial ischemia/reperfusion (MI/R) injury is a common phenomenon in ischemia heart disease [3]. MI/R refers to a heart pathology of reducing the blood perfusion, which lead to reduction of oxygen supply, abnormal myocardial energy metabolism, and abnormal function in the heart [4, 5]. Hence, a better and thorough understanding of myocardial ischemia pathophysiology could lead to significantly improved outcomes in relative treatment.

Currently, noncoding RNA is found to be important in many kinds of disease. Long noncoding RNAs (lncRNAs, >200 nt) are endogenous molecules lacking protein-encoding capacity [6]. There are significant regulators related to many cardiac diseases [7]. Noncoding RNAs such as long noncoding RNAs and circular RNAs have been assessed as potential biomarkers or therapeutic targets for numerous diseases, including cardiovascular diseases [8–10]. In molecular biology, lncRNA could regard to competitive endogenous RNAs (ceRNAs) and compete with other RNAs through miRNA response elements (MREs) [11, 12]. There is a regulatory balance in ceRNA, and when this balance is broken, it will lead to the disorder of life activities and the occurrence of diseases [13–15]. lncRNA Malat1 regulates microvascular function via miR-26b-5p/Mfn1 axis-mediated mitochondrial dynamics [16]. lncRNA Wisper controls cardiac fibrosis and remodeling [17]. Though the significant role of lncRNA on the procession of cardiac disease, the scientific study about the biomolecular mechanism of lncRNA-based ceRNA network in MI/R injury was still need further elucidation [18].

Here, we aimed to construct the lncRNA mediated-ceRNA network to identify key lncRNAs related to MI/R. Enrichment analyses were used to study the function of the differentially expressed genes. Using RT-PCR, lncRNA X-inactive specific transcript (Xist) was identified in the ceRNA network as a potential biomarker of MI/R and verified in a model of hypoxia/reoxygenation (H/R) treatment. This study might contribute a better understanding to the pathogenesis and progression of MI/R injury.

## 2. Method and Materials

### 2.1. Data Acquisition

The RNA-sequencing (RNA-Seq) dataset profile of MI/R and normal tissues (GSE130217) was downloaded from the GEO database (http://www.ncbi.nlm.nih.gov/geo). This dataset included myocardium transcriptome profiles of C57BL/6J mice (3-4 months) in normal physiological and ischemia reperfusion stressed conditions (3 mice per group) [2]. The miRNA profile of microarray which is related to MI/R (GSE124176) was obtained from research of study of Pedretti et al. [19].

### 2.2. Identification of Differentially Expressed Genes (DEGs)

Raw count data of RNA-Seq (GSE130217) including MI/R and normal samples was normalized with DEseq2 *R* package, and differential expressed genes analysis was performed with limma package, which including differentially expressed- (DE-) mRNA (DE-mRNAs), and DE-lncRNA (*p* value <0.05 and FC ≥ 1.1). Besides, affy package was used for normalization of miRNA profile of microarray (GSE124176), and then limma package was used for DE-miRNA analysis (*p* value <0.05 and FC ≥ 1.1).

### 2.3. Function Enrichment Analysis

To uncover the function and underlying mechanism of DEGs, the GO annotations and KEGG pathway analysis were carried out. GO annotations which contained biological process (BP), cellular component (CC) and molecular function (MF), were performed using DAVID database (https://david.ncifcrf.gov) [20, 21]. KEGG network was constructed by Cytoscape ClueGo. The pathways were significant enrichment with *p* value <0.05.

### 2.4. Protein-Protein Interaction (PPI) Network

We imported DEGs into the STRING online database (https://string-db.org) [22] to construct a protein-protein interaction network. Then, we used the Cytoscape v3.6.0 (https://cytoscape.org/) to screen the top upregulated 100 genes in the network as the method in previous studies mentioned [23, 24].

### 2.5. ceRNA Network Construction

We used the starBase website (http://starbase.sysu.edu.cn/) to construct the ceRNA network by predicting miRNA-mRNA and lncRNA–miRNA interaction information [25]. After that, the miRNA that is regulated for both lncRNA and mRNA was selected, and a ceRNA network was constructed using Cytoscape (version v3.6.0).

### 2.6. Cell Culture and Hypoxia/Reoxygenation (H/R) Treatment

Mouse cardiomyocyte HL-1 cells were bought from the American Type Culture Collection (ATCC, Manassas, VA, USA). The cells grew in DMEM (HyClone, South-Logan, UT, USA) with 10% FBS (HyClone) and 1% penicillin-streptomycin (HyClone) in an incubator (5% CO_2_ at 37°C). To establish the H/R model, HL-1 cells were kept in an incubator with 95% N_2_ and 5% CO_2_ for 8 h to experience hypoxia. Then, cells were incubated at a reoxygenation atmosphere of 95% O_2_ and 5% CO_2_ for 16 h. HL-1 cells in normoxia conditions were used as control.

### 2.7. Cell Transfection

Plasmids were purchased from Sangon Biotech (Shanghai). Briefly, HL-1 cells were transfected with the lncRNA Xist overexpression plasmid using Lipofectamine 2000 (Invitrogen, Rockville, MD, USA), while the control group was transfected with the empty plasmid. After 6 hours, the cells were washed and incubated in culture for 48 hours for further analysis.

### 2.8. Apoptosis Assay by Flow Cytometry

HL-1 cells were transfected and cultured for 24 h. Thereafter, the cells were digested, washed, and resuspended in PBS. Finally, the cells were stained using Annexin V-FITC at 4°C in the dark for 20 min and analyzed by flow cytometry. The percentages of apoptosis were detected using flow cytometry (Beckman Coulter, Brea, CA, USA).

### 2.9. Real-Time Quantitative PCR

The commercial TRIzol kit (Invitrogen, USA) was utilized to extract total RNA from HL-1 cells. Thereafter, RNA was reverse-transcribed into cDNAs with a PrimeScript RT Reagent Kit (Takara, Dalian, China). The quantitative experiment was completed using an ABI 7500 PCR instrument (Applied Biosystems, USA) and a SYBR Green Kit (Applied Biosystems, USA), with the relative gene expression levels normalized to GAPDH. Primers are shown in Table 1.

### 2.10. Statistical Analysis

Data are presented as the means ± SD, and comparisons were calculated using Student's *t*-test with GraphPad Prism software.

## 3. Results

### 3.1. Identification of the DEGs in MI/R

**Figure 1:**
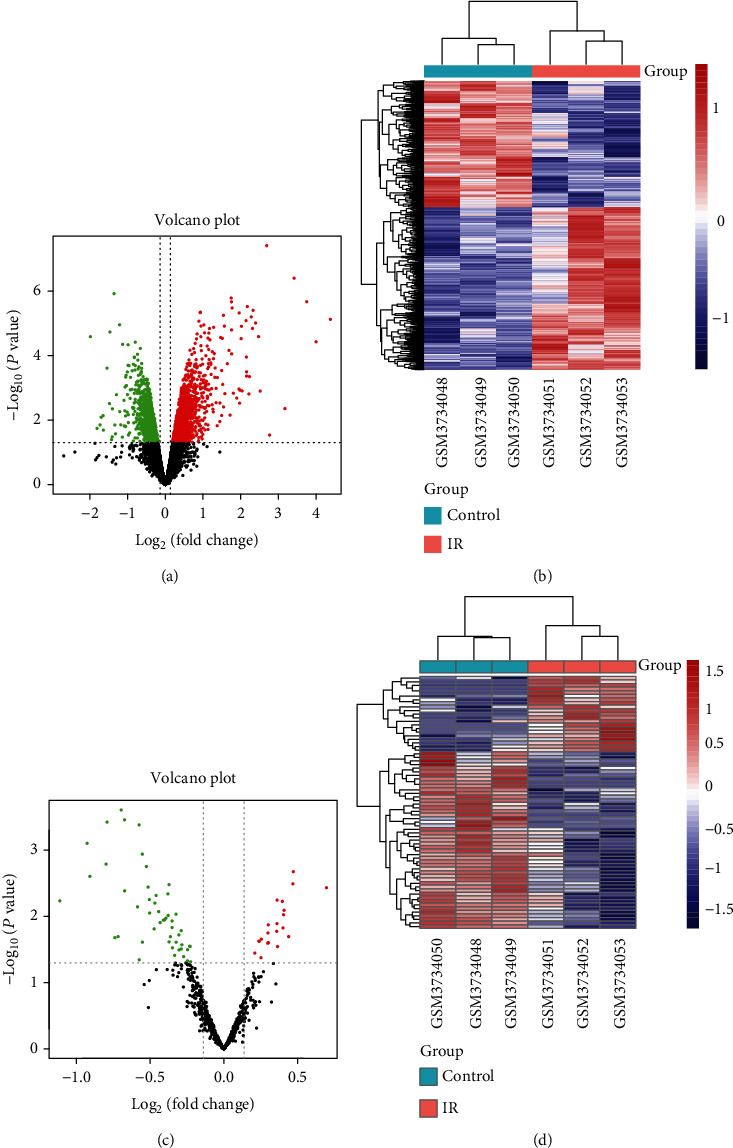
Identification of differential expressed genes (DEGs) in MI/R. (a, b) Volcano plot (a) and heat map (b) of differential expressed- (DE-) mRNAs between MI/R and normal group in dataset GSE130217. (c, d) Volcano plot (c) and heat map (d) of DE-lncRNAs between MI/R group and normal group in dataset GSE130217.

To evaluate the difference in the gene expression between MI/R and normal tissue, DE-mRNAs and DE-lncRNAs were identified based on the GSE137482 dataset. A threshold (*p* value <0.05 and FC ≥ 1.1) was utilized to screen DEGs. As shown in Figure 1(a), a total of 2406 DE-mRNAs were obtained, which includes 1350 upregulated and 1056 downregulated genes. In addition, we also screened out 70 DE-lncRNAs including 21 upregulated and 49 downregulated genes (Figure 1(c)). Otherwise, to have a clearer understanding of the expression distribution of differential genes in the MI/R group and the normal group, we perform heat map cluster analysis on DE-mRNAs and DE-lncRNAs, respectively (Figures 1(b) and 1(d)).

### 3.2. Functional Enrichment Analysis of DEGs

**Figure 2:**
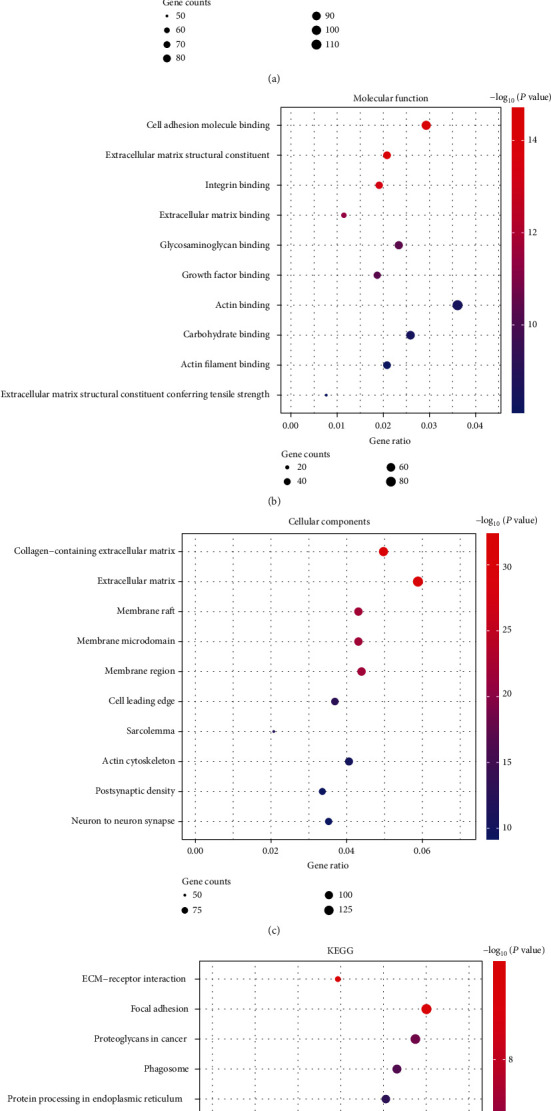
Functional enrichment analyses of the DEGs. (a)–(c) The top 10 enriched biological process (BP) (a), molecular function (MF) (b), and cellular component (CC) (c) of the common DEGs. (d) The KEGG pathway analysis of the common DEGs.

**Figure 3:**
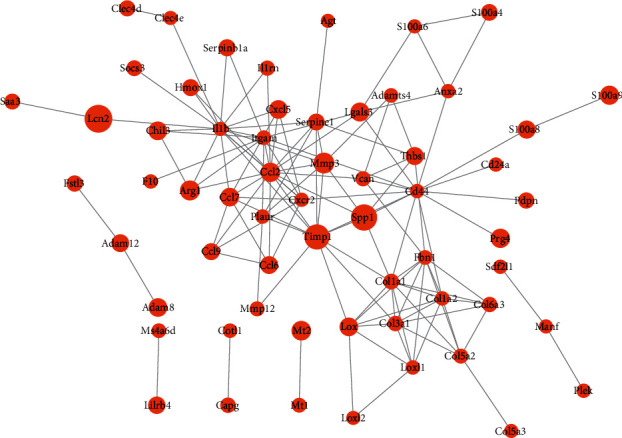
Protein-protein interaction (PPI) network of top 100 upregulated DEGs. The size of the point represents the size of the upregulation, and topological degree was used as the criteria for node size.

To further explore the function and pathways of common DEGs in the biological processes in MI/R, GO and KEGG analysis was performed. The biological processes (BP) (Figure 2(a)), molecular function (MF) (Figure 2(b)), and cell component (CC) (Figure 2(c)) of GO enrichment analysis were presented. The top ten significantly enriched terms including leukocyte migration, cell chemotaxis, extracellular structure/matrix organization, and positive regulation of cell adhesion were significantly enriched in BP (Figure 2(a)), and cell adhesion molecule binding, and extracellular matrix structural constituent were significantly enriched in MF (Figure 2(b)). What is more, we also conducted KEGG pathways analysis and found that lipid and atherosclerosis and ECM − receptor interaction signaling pathways were significantly enriched (Figure 2(d)). These results revealed that these DEGs were related to the pathological process of MI/R. To study the interaction of these of the upregulated DEGs systematically, a PPI network was constructed, and the PPI complex was contained 61 nodes and 126 pairs of PPI relationships (Figure 3).

### 3.3. Construction of an lncRNA-Mediated ceRNA Network

**Figure 4:**
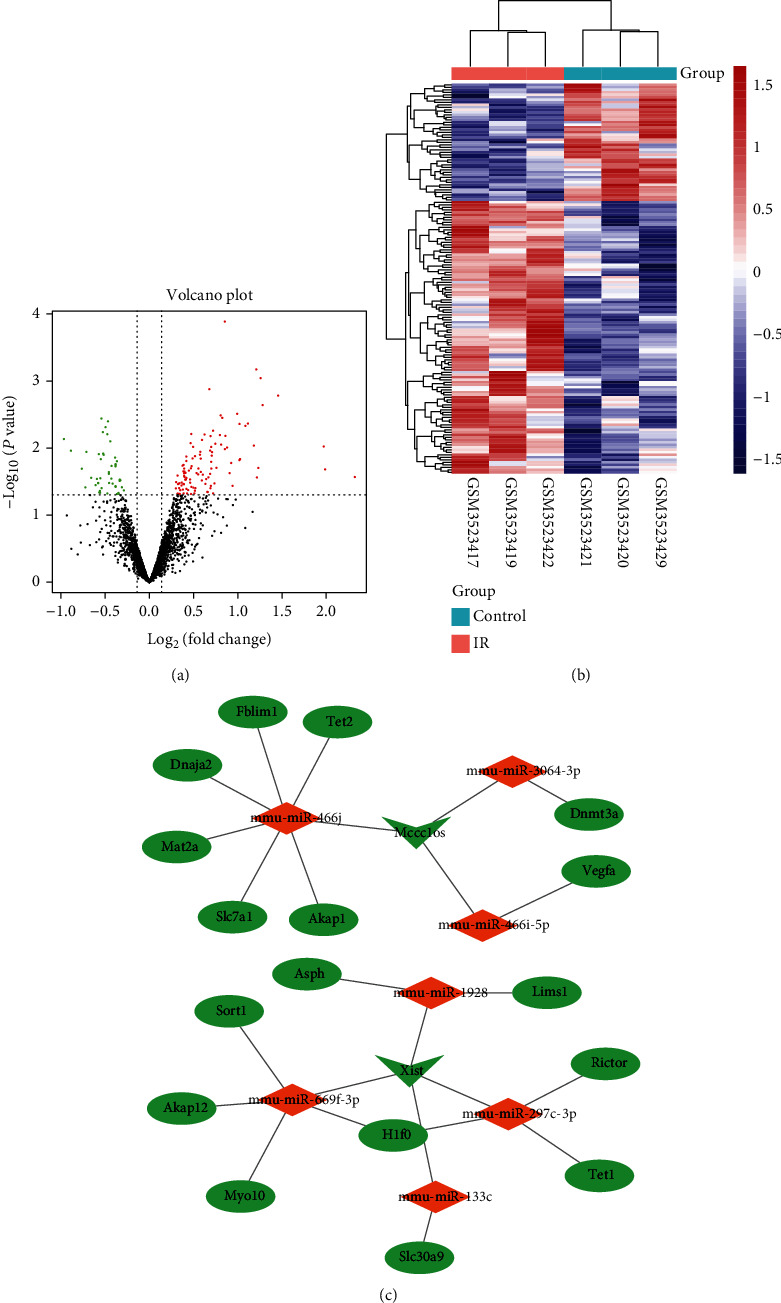
Construction of ceRNA network. (a, b) Volcano plot (a) and heat map (b) of DE-miRNAs between MI/R and normal group in dataset GSE124176. (c) The ceRNA network. In the network, the diamond indicates miRNA, the arrow indicates lncRNA, the oval indicates mRNA, the red means upregulation, and the green means downregulation.

**Figure 5:**
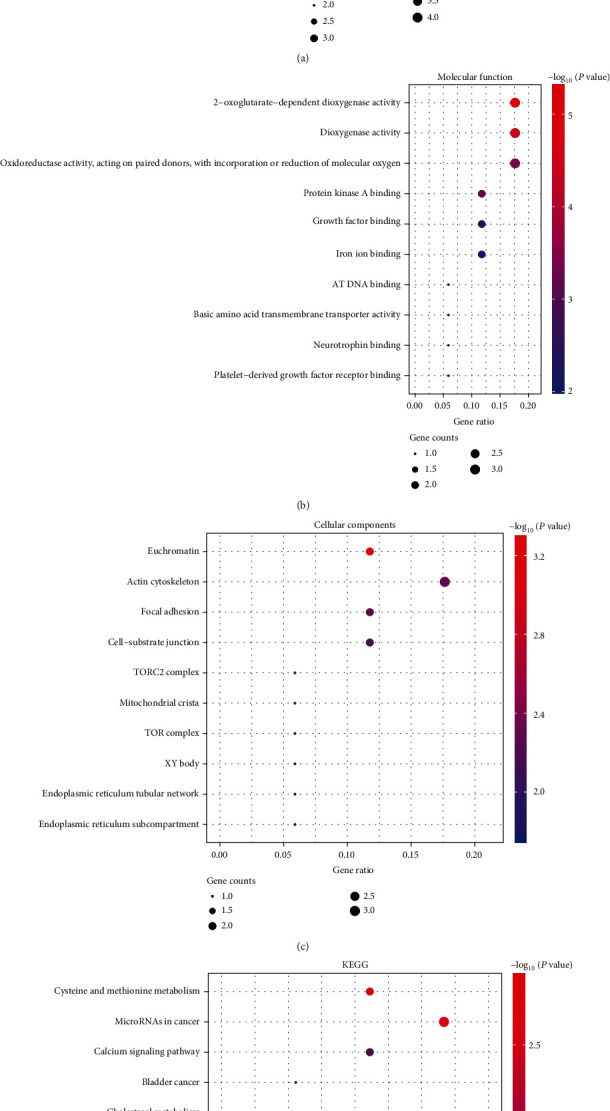
Functional enrichment analyses of the mRNAs in ceRNA network. (a)–(c) The top 10 enriched BP (a), MF (b), and CC (c) of mRNAs in ceRNA network. (d) The KEGG pathway analysis of mRNAs in ceRNA network.

Next, to further explore the interaction between DEGs, an lncRNA-mediated ceRNA network was constructed. 2406 DE-mRNAs and 70 DE-lncRNAs were acquired from previous results. Furthermore, GSE124176 was obtained to identify the expression of DE-miRNAs, and a total of 156 DE-miRNAs were identified, containing 109 upregulated and 47 downregulated DE-miRNAs (Figures 4(a) and 4(b)). Thereafter, a ceRNA network of mRNAs-lncRNAs-miRNAs were constructed using starBase [25]. As shown in Figure 4(c), 17 DE-mRNAs, 7 DE-miRNAs, and 2 DE-lncRNAs were included in the ceRNA network. In the ceRNA network, we found that DE-lncRNA Xist and Mccc1osk were downregulated and can bind to miRNAs in the network. Furthermore, the mRNA involved in the ceRNA network was carried out to KEGG and GO for analyzing the potential function of lncRNA in MI/R. Enrichment analysis enriched DNA methylation and demethylation in BP (Figure 5(a)). Besides, platelet−derived growth factor receptor binding, iron ion binding, oxidoreductase activity, acting on paired donors, with incorporation, and reduction of molecular oxygen were significantly enriched in MF (Figure 5(b)). Actin cytoskeleton was significantly enriched in CC (Figure 5(c)). In addition, KEGG analysis revealed that signaling pathways like VEGF, calcium, cysteine, and methionine metabolism were also significantly enriched (Figure 5(d)). The functional enrichment results indicated that the two lncRNAs play a variety of roles related to the pathological process of MI/R.

### 3.4. Validation of lncRNAs in ceRNA Network

**Figure 6:**
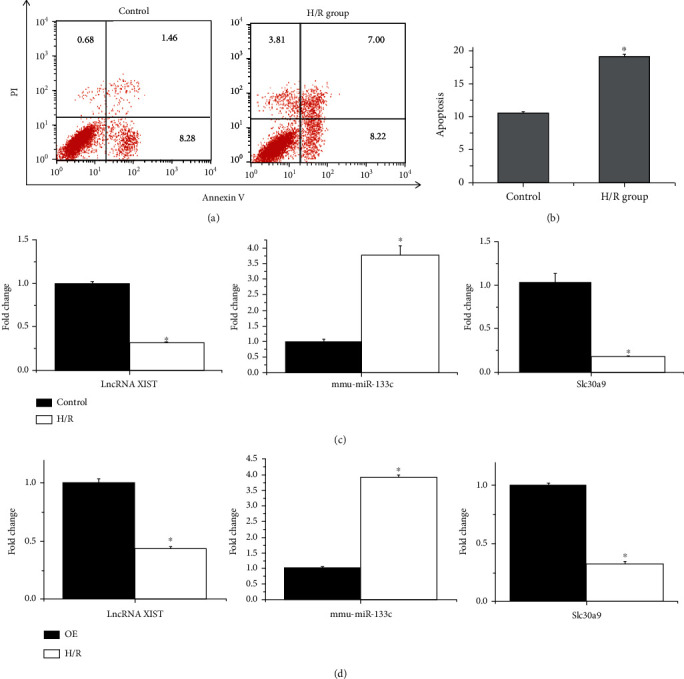
The expression of Xist, slc30a9, and miRNA-133c. (a) The representative images of flow cytometry showing the apoptosis condition of hypoxia/reoxygenation (H/R) treatment HL-1 cell and control group. (b) The apoptosis percentage of H/R and control group. (c) The relative expression level of Xist, slc30a9, and mmu-miR-133c in H/R and control group was detected via qRT-PCR. (d) The relative expression level of Xist, slc30a9, and mmu-miR-133c in H/R and Xist overexpression group was detected via qRT-PCR.

The effect of lncRNA Xist on the MI/R was poorly understood. Therefore, we performed flow cytometry and qRT-PCR for further validation in MI/R. As shown in Figures 6(a) and 6(b), flow cytometry analysis was carried out in HL-1 cells, and apoptosis was accelerated in the H/R group compared with the control group, suggesting that the H/R group was successful modeling (Figure 6(c)). Then, the relative expression of lncRNA Xist was remarkably decreased, while Slc30a9 was also significantly decreased, and mmu-miR-133c was upregulated in H/R model groups compared to control groups. After the overexpression of lncRNA Xist in HL-1 cell, the relative expression of lncRNA Xist was remarkably decreased, while Slc30a9 was also significantly decreased, and mmu-miR-133c was upregulated in H/R model groups compared to overexpression (OE) groups. These results were consistent with the trend presented in the ceRNA network.

## 4. Discussion

As a main cause of cardiac disease-related deaths with high mortality, MI/R injury is mainly cause by coronary heart disease and results in severe myocardial damage [26]. The MI/R injury has caused widespread concern worldwide. Recent studies have indicated that the functional lncRNA-miRNA crosstalk might be a prominent mechanism regulating MI/R injury [27, 28]. In recent years, there are several research suggest that lncRNA-based ceRNA plays a role in MI/R process [29]. For instance, Pei et al. revealed that lncRNA PEAMIR is a ceRNA of miR-29b-3p to suppress apoptosis and inflammatory response MI/R injury [30]. Xue and Luo suggested that lncRNA HIF1A-AS1 is a ceRNA of miRNA-204 to regulate the SOCS2 expression and contributes to ventricular remodeling after MI/R injury [31]. However, detailed mechanism of lncRNA-mediated ceRNA network in pathogenesis of MI/R is needed for further investigation. Here in this study, we analyzed gene expression changes and investigated altered biological processes based on the MI/R related dataset.

MI/R is oxidative stress-related diseases [32], and ischemia-reperfusion injury is caused by some elements including the elevated production of reactive oxygen species, especially at the procession reperfusion [33]. This has been confirmed in several MI/R-related studies [34, 35]. Zhai et al. found that melatonin could ameliorate MI/R injury through regulation of oxidative stress [36, 37]. In our study, GO enrichment analysis also demonstrated that oxidoreductase activity, acting on paired donors, with incorporation and reduction of molecular oxygen, was significantly enriched in MF (Figure 5(b)). This may indicate that the function of the lncRNAs was associated with the regulation of oxidative stress in MI/R. Oxidative stress and inflammation are two primary mechanisms of in MI/R injury and cardioprotection [33]. Otherwise, the physiological myocardial ischemia is linked to Ca^2+^ channel activity [38]. Garcia et al. revealed that inflammasome and ROS produced by Ca^2^+ overload had an effect on mitochondrial function in cardiovascular disease [39]. Also, in our results, the VEGF and calcium signaling pathways were remarkably enriched in the KEGG analysis (Figure 5(d)). In addition, in Figure 5(a), DNA methylation, demethylation, or modification-related terms were enriched. Several studies have shown that lncRNA Xist was associated with DNA modifications including methylation and acetylation [40, 41]. This may indicate that lncRNA Xist may function in MI/R through methylated modification. The detailed functions need further study [42, 43].

lncRNA Xist was reported to be involved in some disease progression, including cerebral ischemia/reperfusion injury [44–46], renal ischemia/reperfusion injury [47, 48], and heart disease [49–55]. These may indicate that lncRNA Xist plays a vital role in I/R injury. Nevertheless, the specific molecular mechanism of lncRNA Xist in MI/R injury is still needed for further investigation. Here in this work, an lncRNA medicated ceRNA network that is related to MI/R was construed. The key lncRNA Xist was identified and verified in H/R-triggered myocardial cells. As an important lncRNA, the lncRNA Xist expression is reduced under H/R conditions, followed by the increased level of miR-133c, thus downregulating the expression levels of Slc30a9. Our study preliminarily confirmed the ceRNA network. Slc30a9 was identified as a mitochondrial zinc transporter [44], which suggests that it might play a role in oxidoreductase activity and echoes our previous functional enrichment results. For the first time, the lncRNA Xist/miR-133c/Slc30a9 axis was identified in H/R-triggered myocardial cells.

In conclusion, in this study, we constructed an lncRNA-mediated ceRNA network based on the DEGs and a subnetwork including Xist/miR-133c/Slc30a9 that was screened out. The functional enrichment analyses revealed that the lncRNAs involved in the ceRNA network might function in oxidative stress and calcium signaling pathway. What is more, we verified the expression level of Xist/miR-133c/Slc30a9 in H/R-triggered myocardial cells. The study might contribute a better understanding to the pathogenesis and progression of MI/R injury and offer a targeted therapy way. However, more features of lncRNA Xist are yet to be studied. Moreover, the Xist/miR-133c/Slc30a9 axis needs further investigation.

## Figures and Tables

**Table 1:** Specific RNA primers for quantitative qRT-PCR analysis.

Gene	Sequencing (5′-3′)
U6	F: CTCGCTTCGGCAGCACATATACT
	R: ACGCTTCACGAATTTGCGTGTC
	RT: ACGCTTCACGAATTTGCGTGTC
mmu-miR-133c	F: CCGCGCGCAAGCTTGTATCTATA
	R: AGTGCAGGGTCCGAGGTATT
	RT: GTCGTATCCAGTGCAGGGTCCGAGGTATTCGCACTGGATACGACCATACC
Slc30a9	F: CAGACATCAGACAGCACATTCC
	R: TAGACCTGGACAGTGGCAATT
lncRNA Xist	F: TTAATTGAGGCGGCAGACTTC
	R: CGATGTTCACCAGTATCTGTTGT
GAPDH	F: ATGGGGAAGGTGAAGGTCG
	R: TCGGGGTCATTGATGGCAACAATA

## Data Availability

The datasets used and analyzed during the current study are available from the corresponding author on reasonable request.
